# Dietary Supplementation of Fructooligosaccharides Enhanced Antioxidant Activity and Cellular Immune Response in Broiler Chickens

**DOI:** 10.3389/fvets.2022.857294

**Published:** 2022-04-14

**Authors:** Tahani Al-Surrayai, Hanan Al-Khalaifah

**Affiliations:** Environment and Life Sciences Research Center, Kuwait Institute for Scientific Research, Safat, Kuwait

**Keywords:** antioxidant, broiler, immune status, prebiotic, cellular response

## Abstract

This study investigated the impact of various concentrations of fructooligosaccharides (FOS) prebiotic on the production performance, antioxidant status, and immune response of broiler chicken. The FOS was used at 0, 0.3, 0.5, and 0.7%. The cycle included 340 broilers distributed into 4 batteries, with 85 broiler chickens in each battery. There were 5 replicates with 17 broiler chickens each, and the analyses were triplicated. The studied parameters were production performance, antioxidant status, hematological measurements, cellular and humoral immune response, intestinal acidosis, intestinal microbial counts, and volatile fatty acid (VFA) level in the hindgut. Results showed that broiler chickens fed 0.7% of FOS had significantly higher body weight gain than the control group and the groups fed 0.3% and 0.5% of FOS. Supplementing broiler feed with FOS at all levels increased the total antioxidant capacity (TAC) and reduced the malondialdehyde of the sera (*P* = 0.015 and 0.025, respectively). Liver catalase enzyme in the broiler chickens fed 0.5 and 0.7% of FOS was higher than that of the control group and the group fed 0.3% of FOS (*P* = 0.001). However, the liver MDA of the control group was higher than that of all the other groups (*P* = 0.031). The total WBC and heterophils % were the highest after supplementing broilers with 0.7% FOS (*P* = 0.004 and 0.003, respectively) at 3 wks of age. Conversely, lymphocytes and monocytes were the lowest for the 0.7% FOS group (*P* = 0.030 and 0.020, respectively). Dietary 0.05 and 0.7% of FOS induced the highest cellular response compared to the other treatments (*P* = 0.020). Thymus, bursa of Fabricious, and spleen weights were enhanced after FOS supplementation, which indicates a higher specific cellular response. To conclude, FOS prebiotic at all levels can be utilized safely to enhance the antioxidant activity and the cellular immune response of broiler chickens. Using 0.7% of FOS resulted in higher body weight of broilers. Accordingly, this amount of FOS is sufficient to reach the required results.

## Introduction

Keeping the health status of poultry flocks at optimum levels is essential to improve the poultry industry and to ensure food security, especially during global pandemics such as the current coronavirus crisis. Using naturally occurring feed ingredients is one way to achieve this goal (1–4). Prebiotics are short-chain oligosaccharide components that are indigestible and trigger the growth and/or activity of beneficial gastrointestinal microbiota in the digestive system. These prebiotics aid in proliferating beneficial bacteria such as *Bacteroides* and *Bifidobacterium* in the gastrointestinal tract (GIT). Some naturally available sources of prebiotics are garlic, onions, and asparagus. Prebiotics contain fiber and oligosaccharides; these influence the amylase production in the GIT, which increases the growth rate of broilers (5–8). Prebiotics are selectively fermented in the colon by native gut microbiota. Prebiotics in the gastrointestinal tract usually target lactic acid bacterial genera *Bifidobacterium* and *Lactobacillus*. The development of these bacterial species has resulted in the production of the bacteriocins, which act against the development of pathogenic microbes such as *Salmonella* sp. and *Escherichia coli*, which improves the health of the chicken (9–15).

Fructooligosaccharide (FOS) is a prebiotic, more commonly found in plants, obtained by enzymatic synthesis through hydrolysis and transglycosylation reactions using sucrose (11). They are claimed to be antioxidants and immunomodulators in both humans and animals (16–21). FOS supplemented in the diets of broiler chickens could improve their growth, immune response, and intestinal mucosa structures, and modulate gut microbiota. The microbiota in the gastrointestinal tract of chickens is crucial for their nutrition, immunity, and physiology. There are few studies involving dietary FOS supplementation and its effects on the immune system of chicken.Biswas et al. (22) investigated the effects of prebiotics (mannan oligosaccharides-MOS and FOS) on physio-biochemical traits, antioxidant and oxidative stability, and carcass traits of 240 commercial broiler chickens. The chickens were fed six iso-caloric and iso-nitrogenous, corn-soya-based dietary treatments. The study team concluded that prebiotics incorporated into the diets of broiler chickens improved their physio-biochemical indices, body weight gain, antioxidant and oxidative stability, and carcass characteristics (22–24). In another study (25), 150 Arbor acres broiler chickens were fed diets supplemented with FOS, and the results showed significant improvement in growth performance and intestinal health, which in turn prevented intestinal damage and enhanced immune response. This suggests that supplementing FOS in broiler chicken diets may be an alternative method to replacing antibiotics and growth promotors.

The objective of the current study is to investigate the effect of different concentrations of FOS prebiotic on the broiler's production performance, antioxidant status, immune response, haemocytometric blood analysis, and intestinal microflora, to determine the optimum concentration to be used for broiler chicken flocks. The hypothesis is that FOS will improve the aforementioned parameters, but the different effects of the different concentrations should be highlighted. Although there are previous studies that investigated the effect of FOS prebiotic on the productive performance parameters in broiler chickens, there is relatively limited data on the direct effect of FOS prebiotic on the antioxidative status of blood and liver tissues in broiler chickens.

## Materials and Methods

### Animal Welfare

This research study was approved by the department committee of the Environment and Life Sciences Research Center in Kuwait Institute for Scientific Research under project No. FA157K (2017). These procedures and protocols followed the official animal welfare guidelines and regulations encoded with reference No. PMO/PV/RP/032/2017. This protocol recommends humane treatment of experimental animals with no pain, stress, or harm.

### Animals, Housing, and Diets

One-day old male Cobb-500broiler chicks were used in the study. The feed and water were provided *ad libitum*. The broilers were fed on a starter diet up to 1 week, on a grower diet up to 2–3 weeks of age, and on a finisher diet for up to 4–5 weeks. The diet was formulatedas per the Cobb 500 guidelines (26) with corn and soya.The broiler cycle involved 340 broilers, distributed into 4 batteries with 85 broilers in each battery. Every battery was comprised of 5 levels, and the area of every level was 0.85 m^2^. In each level, 17 broilers were raised, providing 0.05 m^2^ of space for each broiler chicken. There were four experimental treatments (TRT) used with different levels of FOS (unfortified, 0.3, 0.5, 0.7%). The composition of the basal broiler diet is presented in Table 1. During the initial 3 days of the cycle, 1-day-old chicks were provided light throughout the day to have enough time to find water and feed. The light program was gradually reduced. Artificial bulbs were used as a light source. Minimal vaccination was used for the Cobb-500 broilers as per the guidelines of the supplier. The temperature was kept at 30 °C for 14 d and then gradually reduced to 21 °C by 21 d.

**Table 1:** Chemical composition of the basal broiler diets.

**Ingredient**	**Starter 0–7 d**	**Grower 8–21 d**	**Finisher 22–35 d**
Corn	55.6	57.6	61.2
Soyabean meal	39.4	35.6	32.2
Soya oil	1.3	3.20	3.3
Limestone	1.4	1.45	1.3
Dicalcium Phosphate	1.4	1.4	1.2
Salt	0.2	0.2	0.2
L-Lysine	0.1	0.1	0.1
DL-Methionine	0.27	0.27	0.26
Vit-min premix^*^	0.20	0.20	0.20
Total %	100	100	100
Nutrient composition chemical analysis			
Crude protein (27) (%)	24.0	22.4	21.03
Metabolisable energy (kcal/kg)	2932.2	3054.4	3105.3
Fat (g/kg)	3.86	5.75	6.00
Calculated analysis			
Calcium (g\kg)	0.99	0.98	0.87
Phosphorus (g\kg)	0.41	0.40	0.36
Sodium (g\kg)	0.11	0.11	0.11
Lysine (g\kg CP)	1.45	1.34	1.23
Methionine (g\kg CP)	0.66	0.64	0.61
Choline (mg\kg)	1420.7	1329.4	1260.2

* *Supplied per kg of premix: trans-retinol (A), 12500000IU; cholecalciferol (D3), 500000IU; α-tocopherol acetate (E), 75000 mg; thiamine (B1), 4500 mg; riboflavin (B2), 8000 mg; pyridoxine (B6), 5000 mg; vitamin B12, 22000 mg; pantothenic acid, 20000 mg; folic acid, 2000 mg; biotin, 200000 μg; Fe, 100000 mg; Co, 250 mg; Mn, 100 mg; Cu, 10000 mg; Zn, 80000 mg; I, 1000 mg; Se, 300 mg; Mo, 0.5 mg; Ca, 7.7%; P, 0.01%; Na, 0.18%; Ash, 97%*.

### Sample Collection

Broiler chickens were slaughtered at 35 days of age through stunning and bleeding. Heparinized tubes were used with added liquid heparin to collect blood to avoid clots. Ten broilers per treatment were used for the analyses. Blood samples were collected in triplicates on 3 and 5 weeks of the production cycle. Organ samples like thymic lobes, spleen, fat pads, liver, heart, and bursa of Fabricious were collected in phosphate buffer saline (PBS) for estimating their weights.

### Production Performance Variables in Broilers

The parameters measured were growth rate, feed efficiency, feed consumption, and mortality. Bodyweight and feed consumption were recorded. Temperature and relative humidity were also recorded daily and adjusted accordingly to avoid stressful conditions in the poultry house, especially during summer. Mortality was recorded daily. Broiler chicks were weighed regularly at hatch, after 1 week, and at the end of every 2 weeks, and after that, until the end of the cycle after 35 d.

### Antioxidant Status Measurement

The antioxidant status was investigated by measuring antioxidant indices in sera and livers of the broiler's chickens supplemented with different concentrations of FOS. The liver's antioxidant activity was measured by total superoxide dismutase (TSOD) activity using a Ransod kit from Randox Laboratories, United Kingdom, as described before by Habibiet al. (28). KCL solution at 1.15% was used to homogenize tissue samples. TSOD activity was determined using xanthine and xanthine oxidase to produce a red formazan dye by reaction with 2-(4-iodophenyl)-3-(4-nitrophenol)-5-phenyltetrazolium chloride (INT). TSOD activity was measured by units of the degree of inhibition of the reaction, and the results were expressed as unit/mg protein. Liver glutathione peroxidase (GPx) was indicated based on the protocol of Paglia, Valentine (29). Gpx catalyzes the oxidation of glutathione by cumene hydroperoxide. In the presence of glutathione reductase and NADPH, the oxidized glutathione is immediately converted to the reduced form with concomitant oxidation of NADPH to NADP+. The decrease in absorbance at 340 nm was measured (Ransel kit, Randox Laboratories Ltd. UK). The results of Gpx activity in liver tissue was expressed as unit/ mg protein. The catalase (30) enzyme activity in the liver was determined using the method of Aebi (31). The absorbance of the sample was measured at 240 nm for 30 s in a spectrophotometer. Sera malondialdehyde (32) and total antioxidant capacity (TAC) were measured as described before by Habibi, Sadeghi (28). Ten broilers per treatment were used for this test and samples were analyzed in triplicates.

### Hematological Measurements

This involved evaluating and numerating immune and red blood cells (RBC) using a computerized hemocytometer. Blood samples (about 8–10 ml of blood per tube) were collected from the brachial vein of the chicken in vacutainer tubes (K2EDTA). The samples were kept in an icebox and were instantly analyzed. Total and differential blood quality parameters like hemoglobin (HGB), red blood cells (RBC), white blood cells (WBCs), mean corpuscular hemoglobin concentration (MCHC), hematocrit (HCT), red cell distribution width (RDW), mean corpuscular volume (MCV), thrombocyte platelets count (PLT), and mean corpuscular hemoglobin (MCH) were quantified using cell-Dyn 3,500 haematocytometer (Abbott Laboratories, Abbott Park, IL, USA). Ten broilers per treatment were used for this test.

### Cellular Immune Response

Phytohaemagglutinin (PHA) was dissolved in pyrogen-free PBS. It was injected into the subcutaneous layer of the broiler skin, and subsequent swelling at the injection site (wattle) was measured after 24–72 h, which was interpreted as an index of cell-mediated immunocompetence. In this test, ten chickens at 5 weeks of age were used, as described previously by Goto, Kodama (33) and Corrier and DeLoach (34). The injection site was marked before injection, and the thickness of the injection site was measured by a micrometer. After this, the birds were injected intradermally in the wattle with 0.5 mg of PHA-P in 0.1 ml of PBS. Post injection thickness was typically measured at 24 h post injection, yet 24 h did not reflect the peak of the reaction; it could be measured (to nearest 0.01 mm) at 0, 24, 48, and 72 h after PHA-P injection. Wattle swelling was calculated as the difference between the thickness of the wattle prior to and after the injection of PHA-P.

### Humoral Immune Response

Ten broiler chickens of 5 weeks of age from each treatment were used to test the humoral immune response. Antibody titers were estimated using sheep RBC. The chickens were injected with 1 ml of diluted sheep RBC solution (7 v/v in 0.9% NaCl). After a week of injection, blood serum samples were collected using centrifugation methods, and differential antibody titers were measured using commercial ELISA kits. 50 μl of the respective standards were added to each well in the 96-well tray. Then 40 μl of each sample was added to the sample wells, followed by 10 μl of biotin-conjugated anti-chicken antibody. Then, 50 μl of streptavidin-HRP was added to each sample, carefully avoiding the blank control wells, and reagents were mixed thoroughly. The plate was covered with a sealer and incubated for 60 min at 37°C. After incubation, the sealer was detached, and the plate was washed with wash buffer five times; the wells were overfilled and soaked for at least 30 sec to 1 min (35, 36). After each washing, paper towels were used to blot the plates. Then 50 μl substrate solution A was added to each well followed by 50 μl of substrate solution B (care was taken not to expose the substrate solution B to light as it is light sensitive). The plate was sealed with another sealer and incubated for 10 min in the dark at 37 ^0^C. Simultaneously, after adding 50 μl of stop solution to each well, the blue solution instantly turned yellow. Finally, optical density (OD) value was measured at 40 nm within 30 min, after adding a stop solution using a microplate reader (35).

### Microbial Counts in the Chicken's Gut

Microbial analysis for Lactic acid bacteria (LAB), *Escherichia coli (E. coli*), and *Salmonella* was conducted by extracting the cecum substance as defined by Schoeni and Doyle (37). Ten chicken samples, 35 d of age, were used per treatment. All chicken samples were slaughtered on the farm and transferred to the laboratory under refrigerated conditions for further analysis. In the laboratory, the collected chicken samples were prepared according to the protocol described earlier by Al-Khalaifa, Al-Nasser (10). Each chicken was first weighed and washed with 1:2 diluted disinfectants. The abdominal area was de-feathered and sprayed with 70% ethanol to ensure sterility of the area before dissecting. Then, the skin was cut using a sterile scissor and removed from the abdomen area with sterile forceps. The covering membrane was cut carefully to reach the chicken's digestive system. The lower intestine was surgically exposed, and the caeca were removed aseptically, and their weights were recorded.To isolate the LAB, *Salmonella*, and *E. coli* from the caeca, the contents were extracted as described earlier by Schoeni and Doyle (37). Each caecum's content was squeezed in a sterile petri dish, and then the caeca were split lengthwise with a sterile scalpel and rinsed with 0.85% (w/v) NaCl sterile solution (1:9 v/v) to remove the content. Any residual cecal content was removed gently by scraping the cecal epithelium. The crude extract of the caeca was transferred into a sterile stomacher bag and homogenized for 3 min. The collected crude extracts were used directly for microbial analysis. LAB, *E. coli*, and *Salmonella* counts were determined using standard microbiological methods, as described by Lorch (38) and Al-Khalaifah et al. (1), and the samples were analyzed by applying spreading technology. *E. coli* and *Salmonella*count experiments were conducted using *Brilliance E. Coli* selective and *Xylose-Lysine-Desoxycholate* agar media (Oxoid), respectively, while LAB experiments were conducted using de Man, Rogosa, Sharpe (MRS) media (oxoid). Serial dilution was made from the crude samples, and 0.1 ml of the prepared sample was spread onto the surface of the media with a sterile spreader. The plates were then incubated aerobically for 24 hours at 37 °C for both *E. Coli* and *Salmonella*, whereas for LAB, the plates were incubated anaerobically for 48 hours at 30 °C. The colonies were counted at the end of the incubation periods. The counts were transformed into log values.

### Hindgut Acidosis

Hindgut acidosis represents cecum and/or colon acidity. The pH of the control and experimental treatment groups was measured to indicate broilers' health and their capacity to resist pathogens. This test was done on broilers of 3 and 5 weeks, and ten broiler chickens were used from each treatment. Hindgut digesta was collected into tubes, and pH was measured using a probe.

### Volatile Fatty Acid (VFA) Level in the Hindgut

In screw-capped tubes, around 1.50 g of defrosted digesta was diluted with distilled water (1:1 wt/vol). The solution was homogenized by shaking and centrifuged to collect the clear upper supernatant. The volatile fatty acid profile was analyzed using GC techniques (39), which is a well-established technique for analyzing lipids. The main principle underlying the separation of fatty acids by GC is that the temperatures under which they become volatile differs. This temperature depends on the carbon chain length and the number and position of double bonds in the molecules. Before analyzing the samples, the machine was standardized by using the standard mixes of short-chain volatile fatty acid. All of the required fatty acid peaks were obtained and standardized. This test was done on broilers of 3 and 5 weeks of age, and ten broiler chickens were used in each test.

### Statistical Analyses

One-way ANOVA and the general linear model method were used to analyze the overall differences between the treatments, and the analysis was done with Minitab software (Minitab Inc., State College, PA). The difference in treatment means was considered significant at *P* < 0.05 using parametric study and Bonferroni test was used.

## Results

### Production Performance Parameters

Table 2 shows the effects of different levels of FOS on the body weight, feed consumption, feed efficiency, and weekly body weight gain of broiler chickens. The different levels of FOS had a significant effect on the bodyweight of 1- and 2-wks-old broilers. Results in Table 2 show that using FOS significantly improved body weight at 1 and 2 wks of age, regardless of FOS level. However, there was no significant effect of FOS supplementation on the bodyweight of broilers at 3 and 4 wks of age. At 5 wks of age, feeding 7% of FOS improved the body weight, compared to the other dietary treatments (Table 2). Feed consumption was not affected by the different FOS treatments at all ages. There was no effect of the different levels of FOS on the feed efficiency of broilers at all ages. Results of Table 2 also show that at 2–5 wks of age, birds fed 0.7% of FOS had significantly higher weekly body weight gain than the control group and the groups fed 0.3 and 0.5% of FOS.

**Table 2:** The effects of different levels of FOS on the body weight, feed consumption, feed efficiency, and weekly body weight gain of broiler chickens.

	**FOS %**					
**Age**	**Control**	**0.3**	**0.5**	**0.7**	**SEM**	**P-value**
Body weight						
1 d	41.0	40.0	41.0	42.0	0.000	-
1 wk	109.0^a^	120.0^b^	125.0^b^	124.0^b^	2.252	0.001
2 wk	240.0^a^	255.0^b^	270.0^bc^	275.0^c^	3.645	0.001
3 wk	655.0	657.0	667.0	669.0	25.51	0.451
4 wk	1034.0	1035.0	1044.0	1056.0	10.40	0.551
5 wk	1590.0^a^	1585.0^a^	1605.0^a^	1760.0^b^	11.02	0.025
Feed consumption						
1 wk	200.0	200.1	201.0	201.1	1.105	0.77
2 wk	299.0	300.20	302.32	300.00	2.178	0.98
3 wk	498.21	500.00	510.11	506.00	4.554	1.000
4 wk	790.0	785.00	780.01	789.32	5.477	0.645
5 wk	860.0^a^	865.0^a^	850.0^a^	890.0^b^	15.0	0.046
Feed Efficiency						
1 wk	2.90	2.85	2.62^a^	2.80^b^	0.067	0.868
2 wk	1.87	1.70	1.83	1.83	0.098	0.345
3 wk	1.30	1.25	1.31	1.31	0.021	0.876
4 wk	2.19	2.09	2.14	2.19	0.064	0.908
5 wk	1.70	1.70	1.65	1.61	0.059	0.596
Weekly body weight gain						
1 wk	70.00	70.01	76.55	72.00	3.154	0.412
2 wk	160.00^a^	175.65^b^	165.00^a^	164.00^a^	3.940	0.003
3 wk	387.00^a^	399.00^b^	389.00^a^	386.23^a^	2.120	0.005
4 wk	360.11^a^	375.0^b^	364.00^a^	361.00^a^	4.140	0.003
5 wk	503.0	508.0	513.0	550.0	50.01	0.278

*FOS, Fructo-oligosaccharides. All treatment groups received a soybean basal diet. Differences between the treatment groups are statistically different at P < 0.05. Mean values within the same raw with different superscript letters are significantly different*.

### Antioxidant Status

Table 3 shows the effects of various concentrations of FOS on antioxidant indices in serum and liver tissues of the broilers. The results show that broilers fed on 0.5 and 0.3% of FOS showed significantly higher total antioxidant capacity (TAC) in serum than broilers fed diets containing 0.7% of FOS (*P* < 0.05). On the other hand, malondialdehyde (32) in the sera of broilers fed diets containing 0.5 and 0.3% of FOS was significantly lower than that of broiler chickens fed diets with 0.7% of FOS (*P* < 0.05). There was no significant difference in the total superoxide dismutase (TSOD) and liver glutathione peroxidase (GPx) of livers of broiler chickens fed the different dietary treatments. The Catalase enzyme (30) values were significantly higher with 0.5% and 0.7% FOS compared with that with control (*P* < 0.05). However, the livers of broilers fed 0.5% of FOS had significantly higher MDA than those fed 0.7% of FOS, which is significantly higher than those fed 0.3% of FOS (Table 3).

**Table 3:** Antioxidant parameters and malondialdehyde (32) in serum and livers of 5-wks-old broiler chickens fed various concentrations of FOS.

**Parameter**	**FOS treatment %**					
	**Control**	**0.3**	**0.5**	**0.7**	**SEM**	***P*** **-value**
Serum						
TAC (mmol/l)	0.39^a^	0.9^b^	1.01^b^	0.5^c^	0.02	0.015
MDA (nmol/ml)	5.12^a^	2.1^b^	2.5^b^	3.8^c^	0.04	0.025
Liver						
TSOD (U/mg pro.)	3.99	4.0	4.0	4.1	0.45	0.452
GPx (U/mg pro.)	0.68	0.60	0.70	0.70	0.09	0.847
CAT (k/mg pro.)	0.30^a^	0.30^a^	0.80^b^	0.70^b^	0.02	0.001
MDA (nmol/mg pro.)	8.01^a^	3.7^b^	7.1^c^	5.01^d^	0.11	0.031

*FOS, Fructo-oligosaccharides. All treatment groups received a soybean basal diet. Differences between the treatment groups are statistically different at P < 0.05, TAC, Total Antioxidant Capacity; MDA, Malondialdehyde; TSOD, Total Superoxide Dismutase; GPx, Liver Glutathione Peroxidase; CAT, Catalase enzyme. Mean values within the same raw with different superscript letters are significantly different*.

### Hematological Measurements

All broilers seemed healthy, and no mortality was observed. Table 4 shows the impact of the various concentrations of FOS on the blood composition of broiler chickens at 3 and 5 wks of age. Significant differences were observed in the white blood cells (WBC), heterophils, lymphocytes, and monocytes of 3-wk-old chickens (*P* < 0.05). Results in Table 4 show a significant difference in the total WBC after feeding broilers a diet supplemented with 0.7% of FOS at 3 wks of age. The percentage of heterophils was the highest for the treatment group fed 0.7% of FOS and the lowest for the group fed 0.3% of FOS.

**Table 4:** Biochemical and hematological parameters of 3- and 5-wk-old broilers feddifferent concentrations of FOS.

	**FOS treatment g/kg**											
**Parameter**	**Control**	**0.3%**	**0.5%**	**0.7%**	**SEM**	***P*** **-Value**	**Control**	**0.3%**	**0.5%**	**0.7%**	**SEM**	***P*** **-Value**
	**3-wks of age**						**5-wks of age**					
WBC (K/uL)	22.52^a^	21.80^a^	23.22^a^	41.22^b^	5.04	0.004	23.85	22.32	25.09	25.01	6.00	0.632
Heterophils (%)	42.51^a^	43.04^a^	41.00^a^	60.30^b^	4.03	0.003	31.95	35.14	30.23	33.12	4.65	0.601
Lymphocytes (%)	30.98^a^	35.70^a^	30.10^a^	5.12^b^	5.12	0.030	40.98	40.12	41.27	43.03	5.32	0.409
Monocytes (%)	14.85^a^	11.90^a^	17.53^a^	1.11^b^	3.54	0.020	12.99	13.00	13.21	14.12	0.99	0.509
Eosinophils (%)	2.84	2.99	2.0	2.28	1.1	0.212	0.041	0.09	0.05	0.10	0.09	0.465
Basophils (%)	4.01	3.32	4.11	3.99	1.30	0.121	5.02	5.04	4.32	5.00	1.00	0.783
RBC (M/uL)	4.21	4.31	4.20	4.00	0.20	0.190	4.02	3.09	4.09	2.99	0.15	0.203
HGB (g/dL)	16.89	17.32	17.87	17.00	1.02	0.090	20.01	16.98	19.09	20.00	0.93	0.304
HCT%	40.84	42.98	43.00	41.20	2.66	0.110	40.99	39.00	41.33	45.43	3.15	0.209
MCV (fL)	135.98	130.99	141.32	134.43	3.12	0.212	125.98	130.03	120.21	132.00	2.99	0.523
MCH (pg)	50.99	50.11	54.44	52.12	0.51	0.090	50.32	53.40	50.32	56.00	0.70	0.243
MCHC (g/dL)	42.15	42.12	40.00	43.43	0.543	0.423	40.98	40.24	40.97	41.00	1.09	0.098
RDW%	12.13	11.63	13.00	11.99	0.424	0.067	11.98	11.37	12.09	11.08	1.64	0.492
PLT (K/uL)	6.15	6.12	6.06	5.99	2.59	0.315	6.98	6.92	8.98	4.98	1.02	0.432

*FOS, Fructooligosaccharides. Means within rows are significantly different at p < 0.05, n = 10; SEM, Standard error of the mean; WBC, white blood cells; RBC, red blood cells; HGB, haemoglobin; HCT, haematocrit; MCV, mean corpuscular volume; MCH, mean corpuscular hemoglobinhaemoglobin; MCHC, mean corpuscular hemoglobin haemoglobin concentration; RDW, red cell distribution width; PLT, thrombocyte platelet count. Mean values within the same raw with different superscript letters are significantly different*.

Conversely, the percentage of lymphocytes was the lowest for the group fed a diet rich in 0.7% of FOS, and the percentage of monocytes was the lowest for the group fed the control diet (CO). The percentage of eosinophils was higher in the group fed a diet supplemented with 0.7% of FOS than that in any other group. There is no significant impact on the percentage of basophils, red blood cells (RBC), hemoglobin (HGB), haematocrit (HCT), mean corpuscular volume (MCV), mean corpuscular hemoglobin (MCH), mean corpuscular hemoglobin concentration (MCHC), red cell distribution width (RDW), and platelet count (PLT) among the broiler groups. The results showed no significant effect on blood biochemical parameters among the different treatment groups of 5 wks of age (Table 4).

### Cellular Immune Response

Table 5 shows the effects of various dietary treatments on wattle swelling changes of 3- and 5-wk-old broiler chickens. The results showed that supplementation of 0.7% FOS resulted in significantly lower cellular response in 3-wk-old broiler chickens, compared to the other treatments (*P* = 0.020). There was no significant effect on wattle swelling in broilers at 5 wks of age.

**Table 5:** Effect of different dietary treatments of 3- and 4-wk old broilers on wattle swelling.

	**Treatments**					
	**Control**	**0.3%**	**0.5%**	**0.7%**	**SEM**	***P*** **-value**
3-wk-old broilers Thickness (mm)	1.00^a^	2.13^b^	1.98^b^	1.03^a^	0.375	0.020
4-wk-old broilers Thickness (mm)	2.01	2.20	2.01	2.34	0.512	0.231

*FOS, Fructooligosaccharides. Soybean basal diet has been supplemented for all treatment groups. The difference in treatment means among the groups is statistically different at P < 0.05. Mean values within the same raw with different superscript letters are significantly different*.

### Humoral Immune Response

Table 6 shows the effect of different FOS concentrations on the antibody titers of broiler chicken at 3- and 5-wks of age. No significant effect was observed.

**Table 6:** Effect of various FOS concentrations on antibodies of 5-wk-old broiler chickens.

**Treatment**	**Antibody concentration**		
	**IgY**	**IgA**	**IgM**
Control	0.38	0.10	0.008
FOS (0.3%)	0.48	0.10	0.011
FOS (0.5%)	0.41	0.12	0.014
FOS (0.7%)	0.39	0.11	0.007
SEM	0.170	0.024	0.002
*P*-value	0.901	0.913	0.493

*FOS, Fructooligosaccharide; IgM, Immunoglobulin M; IgY, Immunoglobulin Y; IgA, Immunoglobulin A. The difference in treatment means among the groups is statistically different at P < 0.05*.

### Microbial Counts and Hindgut Acidosis

Table 7 shows the effect of different concentrations of FOS on the microbial counts of broiler chickens at 3- and 5-wks of age. The results show no significant difference in the microbial counts. Salmonella count was not observed in all the treatments, and the pH tended to have become acidic from alkaline in all the treatments. There was, however, a borderline effect of the different FOS levels on the pH value of the intestinal solution (*P* = 0.051). If this borderline effect is considered, broiler chickens fed a diet with 0.7% of FOS showed the lowest pH value, which means the highest acidity in the intestine. Chickens use this acidity as a means to get rid of pathogens (Table 7). The results also show no statistical difference, and salmonella counts were not recorded in all the dietary treatments of broilers at 5 wks of age.

**Table 7:** Effect of various concentrations of FOS on microbial count in 3- and 5-wk-old broiler chickens.

	**Treatment (FOS %)**											
	**Control**	**0.3**	**0.5**	**0.7**	**SEM**	***P*** **-value**	**Control**	**0.3**	**0.5**	**0.7**	**SEM**	***P*** **-value**
**3-wks of age**						**5-wks of age**						
Log LAB	7.96	7.63	7.24	7.93	0.137	0.247	6.53	6.53	6.61	6.96	0.202	0.321
Log *E.coli*	7.30	7.46	6.98	6.66	0.341	0.290	6.16	6.00	5.74	6.25	0.315	0.124
pH	6.80	5.95	6.08	5.51	0.203	0.051	6.52	6.13	6.02	6.53	0.200	0.120

*FOS, Fructooligosaccharides; Difference in treatment means among the groups is statistically different at P <0.05*.

### Volatile Fatty Acid Profile in the Hindgut

Table 8 shows the short fatty acid composition of broilers at 3- and 5-wks-old broilers fed various levels of FOS prebiotic. The results show no significant effect.

**Table 8:** Effect of different concentration of FOS on the composition of short fatty acid of 3- and 5-wk-old broilers.

	**Treatments (FOS %)**											
**Fatty acids**	**Control**	**0.3**	**0.5**	**0.7**	**SEM**	***p*** **-value**	**Control**	**0.3**	**0.5**	**0.7**	**SEM**	***p*** **-value**
	**3-wks of age**						**5-wks of age**					
Acetic acid	1.12	33.16	0.92	14.22	18.300	0.498	52.60	62.14	60.89	62.92	16.25	0.885
Propionic acid	0.02	0.02	0.00	0.09	0.053	0.519	0.00	0.00	0.00	0.00	0.00	0.00
Butyric acid	0.00	3.74	0.00	0.48	1.207	0.135	3.41	0.90	1.12	1.52	1.05	0.290
Isovaleric acid	0.00	0.00	0.00	0.07	0.04	0.422	0.00	0.00	0.00	0.00	0.00	0.00
Valeric acid	0.01	0.00	0.13	0.15	0.117	0.629	0.00	0.00	0.00	0.00	0.00	0.00
Total	1.15	36.94	1.06	15.03	17.23	0.391	56.01	63.04	62.45	64.44	16.25	0.927

*FOS, Fructooligosaccharides; Soybean basal diet has been supplemented for all treatment groups. The difference in treatment means among the groups is statistically different at P < 0.05*.

### Immune Tissue Weight

The effect of different concentrations of FOS on the weight of the immune tissue of 3- and 5-wk-old broilers has been studied (Table 9). The results in Table 9 show that supplementing 3-wk-old broiler chickens with FOS at different concentrations significantly enhanced the weights of the broilers' spleen, bursa of Fabricius, and thymus (*p* = 0.030, 0.045, and 0.027, respectively). Conversely, feeding the broiler chickens different concentrations of FOS showed no significant effect on the tissue weight of their different organs (Table 9).

**Table 9:** Effect of different concentration of FOS on tissue weight in 3- and 4-wk-old broiler chickens.

**Diet**	**Abdominal fat pad**	**Heart**	**Spleen**	**Liver**	**Bursa of fabricius**	**Thymus**	**Breast**	**Leg+Thigh**
**% Body weight**								
**3-wk-old broiler chickens**								
Control	0.61	0.60	0.05^a^	3.54	0.10^a^	0.09^a^	7.25	5.12
0.3%	0.66	0.52	0.09^c^	3.18	0.20^b^	0.38^b^	6.9	6.7
0.5%	0.62	0.58	0.15^b^	3.34	0.21^b^	0.32^b^	6.04	4.43
0.7%	0.62	0.70	0.15^b^	3.96	0.22^a^	0.11^c^	9.41	6.82
SEM	0.19	0.09	0.01	0.39	0.02	0.05	1.43	1.22
*P*-value	0.986	0.381	0.030	0.390	0.045	0.027	0.298	0.357
**4-wk-old broiler chickens**								
control	1.10	0.52	0.19	2.99	0.10	0.29	5.98	3.99
0.3%	0.91	0.57	0.16	3.53	0.09	0.36	4.94	4.22
0.5%	0.61	0.56	0.20	3.58	0.13	0.23	6.79	4.00
0.7%	1.03	0.50	0.14	3.16	0.09	0.31	6.76	4.91
SEM	0.33	0.04	0.03	0.24	0.01	0.08	0.52	0.48
*P*-value	0.668	0.477	0.307	0.46	0.231	0.554	0.073	0.428

*FOS, Fructooligosaccharides; Soybean basal diet has been supplemented for all treatment groups. The difference in treatment means among the groups is statistically different at P < 0.05. Mean values within the same column with different superscript letters are significantly different*.

## Discussion

This study aimed to investigate the effect of various concentrations of commercially developed FOS prebiotic on the productive performance, immune response, and oxidative activity of broiler chickens. Various FOS concentrations (0, 0.3, 0.5, and 0.7%) of the total diet were used. The results of proximate analysis of broiler feed showed normal ranges of fat and protein in the rations. No extreme mortality, outbreaks, or abnormal growth were noticed.

Studies in the literature suggest positive effects of prebiotic supplementation such as FOS on the productive performance parameters of broiler chickens, including their bodyweight. For example, Ammerman et al. (40) investigated the effect of supplementing broiler chickens with FOS at 0.375% on body weight gain and breast weight. The authors revealed that, compared to the control group, FOS supplementation enhanced the weights of broilers at 47 d of age, improved percentage carcass and breast weights, and reduced percentage fat pad. This is in line with the current results that showed a significant gain in the body weight of broiler chickens fed 0.7% of FOS than that of the control group and the groups fed 0.3 and 0.5% of FOS. Similarly, GAO, ZHOU (41) also investigated the effects of FOS on broiler chickens and observed that FOS significantly increased their body weight and feed conversion ratio. However, excessive FOS supplementation (i.e., 1%) may lead to negative impacts such as diarrhea; it can also produce carbon dioxide and hydrogen gases because of fermentation in the GIT, which leads to a decline in the broilers' productive performance (42). However, in contrast to our study, a study by Shang et al. (43) found no significant effects of FOS on the growth performance of Ross × Ross broiler chicks. This may indicate a different effect due to differences with different genetic strains.

Commercial broiler chickens should be raised in controlled environments with ideal management practices to achieve minimal stress. Environmental stress in chickens forms free oxygen radicles from the various metabolic pathways. This leads to oxidative stress and damage to the normal biological functions of their body. This oxidation effect can be lowered by the antioxidant enzyme actions such as TSOD, GPx, and CAT (28, 44). The important enzymes which have antioxidative stress function to eliminate free radicals are GPx and SOD. They have an inter-protective effect which helps in maintaining a healthy balance between oxidants and antioxidants. MDA is a direct product of lipid peroxidation produced after a radical attack on unsaturated fatty acids. It is a crucial indicator of lipid peroxidation level and also as an indirect reflection of cell damage. MDA may be responsible for DNA fragmentation, cell memebrane strucrure destruction, cross-linking and apoptosis. The estrimation of GPx and SOD acitivity and MDA content gives the oxygen free radical level (45). Dietary effective ingredients such as some herbs, synthetic antioxidants, prebiotics, probiotics, and synbiotics are known to elevate the level of antioxidant enzymes that get rid of the free radicals in the body (46).

Studies in the literature have shown that the FOS had been used as an antioxidant and an immunomodulator in humans and animals. The antioxidant status against free radicals is represented by TAC, TSOD, GPx, CAT, and MDA concentrations in the sera and livers of broilers chickens. In the current study, the higher TAC and the lower MDA levels in the sera of FOS-supplemented broilers indicate higher antioxidant status in the supplemented groups than the control group.TAC accurately represents the antioxidant status of an organism. It also gives a precise measurement of the antioxidant status of an organism. There exists a balance between the production of free radical and antioxidants, but due to stressful conditions, there is a shift in balance toward free radicals and oxidative stress is developed, which can be harmful to cellular machinery, enzymes and DNA and protein (47). Levels of TSOD and GPx enzymes were similar among the three dietary levels of FOS. Feeding broiler chickens on a diet containing FOS induced more antioxidant status than feeding chickens on diets with no FOS. The present study also indicated that the highest liver antioxidant status was observed in broilers supplemented with FOS prebiotic. The results of this study revealed that using FOS in the diet of the broiler chickens induced antioxidant reactions in blood and livers, indicated by antioxidant enzyme action.Similarly, (45) et al. studied the effect of a synbiotic on antioxidative status of broiler blood. Their results showed a significant reduction in MDA concentration in comparison to control. There was a significant increase in GPx activity and significant decrease in MDA in control compared to experimental treatments. It is an indicator that nutrition is key to maintaining the pro-oxidant and antioxidantbalance. Increase in CAT, GPx and glutathione reductase was also observed. This could be due to age, colonization resistance, and susceptibility to environmental pathogens of broilers.

Interestingly, some studies have shown that using FOS with probiotics improved the antioxidative status in chickens. For example, Mohammed et al. (16) reported that using a synbiotic supplement (probiotic made up of 4 microbial strains and prebiotic- fructooligosaccharides) improved the antioxidant status of Ross 708 broiler chicks. Mohammed et al. (48) also studied the effects of using synbiotics (4 microbial strains of probiotic and FOS) in broiler chicks reared under heat stress. They observed that the synbiotic improved the antioxidant status and inhibited the harmful effects of heat stress on broilers. In addition, Popović et al. (45) reported that a synbiotic supplementation (*Enterococcus faecim* + fructooligosaccharides), when used in the diets of day-old chickens, improved the blood antioxidant activity of broiler chickens. Furthermore, Li et al. (49) used chitooligosaccharide (COS), a natural alkaline polymer of glucosamine, as a prebiotic in feed rations of 1-day-old Arbor Acres broiler chicks. The authors revealed that COS has an antibacterial effect that regulates lipid metabolism and promotes antioxidant activity and immunity. The mechanisms by which synbiotics can positively impact the antioxidant status of poultry are still not clearly defined. A possible explanation could be that synbiotic promotes scavenging reactive oxygen species (ROS), which inhibits lipid peroxidation and thus promotes antioxidant capability through activation and translocation of nuclear factors, which results in the expression of certain enzymes in the antioxidant defense system (48).

In the current study, dietary 0.7 % of FOS induced the lowest cellular response compared to the other dietary treatments (*P* = 0.020), represented by the wattle swelling. The significant effects of feeding broilers different levels of FOS on tissue weight indicated the immunomodulation effect of FOS on the specific cellular response of broilers, represented by T- and B- cells. The effect of FOS concentrations on antibody titers of 5-wk-old broiler chickens was insignificant.

The hindgut pH was driven toward acidity in all of the FOS-supplemented treatments; however, this was insignificant as it was the only numerical difference. The increase in acidity in the hindgut is due to the short volatile fatty acids like propionic, acetic, lactic, and butyric acids. Antimicrobial agents are also produced in the intestine, which eliminates the pathogenic agents. These substances act against pathogenic agents and exclude them.

The results of the current investigation agree with the previously reported literature. For example, Wang et al. (50) conducted a study on 108 Arbor acres broiler and supplemented their diets with microencapsulated probiotics and prebiotics (MEP), which included 250 mg/g FOS. They found that MEP significantly increased the growth performance, antioxidative abilities, immune functions, and caecal microflora of chickens. In addition, Biswas et al. (51) showed that using monooligo saccharides prebiotic (52) at 0.2% of the total diet can replace antibiotics and act as a growth promoter and immune system stimulant when compared to the control group that was fed a diet containing bacitracin methyl di-salicylate antibiotics. Besides, Wang et al. (50) fed broilers a diet supplemented with microencapsulated probiotics and prebiotics and studied their effects on the immune status. The results revealed that using probiotics and/or prebiotics in the diet of broilers increased serum immunoglobulin M on day 21 and serum total antioxidant capacity level on day 42, relative to the control group. The counts of Lactobacillus in the caeca were also improved by adding pro-/prebiotics (50). In addition, Mookiah et al. (53) investigated the effects of prebiotics, probiotics, and synbiotics on the growth parameters and fatty acid profile in the broiler caeca. They showed that using prebiotics, probiotics, and/or synbiotics improved growth and increased the levels of *Lactobacilli* and *Bifidobacteria* in their caeca. E. coli was decreased on day 21. In the same study, the short volatile fatty acids were increased in the caeca on days 21 and 42, indicating immune status improvement (53).

The gut microflora of young birds is unstable and can easily be affected by infection through pathogens. Therefore, maintaining an optimal gut microflora is a key factor in determining the health and growth of the bird. FOS can selectively stimulate growth and can activate the metabolism of beneficial bacteria such as the Lactic acid bacteria, and can also inhibit the growth of pathogens, ultimately boosting the host's microbial balance. FOS as a dietary supplement is also known to improve the growth performance of broiler chickens (54).

Our study showed that the effect of different concentrations of FOS on the blood biochemical and hematological parameters of broilers was insignificant (*P* ≥ 0.0.5). However, this is in contrast to the findings of Makii (55) as they studied the effects of FOS on humoral immunity induced by infectious bursal disease (IBD) vaccine and some hematological parameters of broilers with and without feeding an aflatoxin-contaminated diet. They observed that FOS improved the immune response and minimized the adverse effects of aflatoxin on some hematological parameters of the chickens.

Studies have been reported demonstrating the positive effects of dietary FOS on the modulation of the immune system of broiler chickens. Birds fed with FOS have been observed to show reduced percentage of B cells and a depressed mitogen response of lymphocytes in the cecal tonsil without having any negative impact on growth performance. A change in number of heterophils and lymphocytes is an indicator of stress in poultry. Generally, the heterophil count increases whereas the lymphocytes decrease (43). Lymphocytes play a key role in humoral antibody formation and cellular immunity. An increase in lymphocyte percentages due to addition of prebiotics, probiotics or synbiotics is indicative of an immuno-stimulatory effect. Probiotics and prebiotics can trigger a protective immune response and thus improve resistance to microbial pathogens in broiler chickens (4, 27, 56).

## Conclusion

In conclusion, using FOS prebiotic at 0.7% significantly enhanced the body weights of broiler chickens. FOS prebiotic at all levels enhanced the antioxidant activity and the cellular immune response of broiler chickens.

## Data Availability Statement

The original contributions presented in the study are included in the article/supplementary material, further inquiries can be directed to the corresponding author.

## Ethics Statement

This research study was approved by the department committee of the Environment and Life Sciences Research Center in Kuwait Institute for Scientific Research under project No. FA157K (2017). These procedures and protocols followed the official animal welfare guidelines and regulations encoded with reference No. PMO/PV/RP/032/2017.

## Author Contributions

All authors listed have made a substantial, direct, and intellectual contribution to the work and approved it for publication.

## Conflict of Interest

The authors declare that the research was conducted in the absence of any commercial or financial relationships that could be construed as a potential conflict of interest.

## Publisher's Note

All claims expressed in this article are solely those of the authors and do not necessarily represent those of their affiliated organizations, or those of the publisher, the editors and the reviewers. Any product that may be evaluated in this article, or claim that may be made by its manufacturer, is not guaranteed or endorsed by the publisher.
